# The roles of platelet-derived growth factors and their receptors in brain radiation necrosis

**DOI:** 10.1186/1748-717X-9-51

**Published:** 2014-02-11

**Authors:** Tomo Miyata, Taichiro Toho, Naosuke Nonoguchi, Motomasa Furuse, Hiroko Kuwabara, Erina Yoritsune, Shinji Kawabata, Toshihiko Kuroiwa, Shin-Ichi Miyatake

**Affiliations:** 1 Department of Neurosurgery, Osaka Medical College, 2-7 Daigaku-machi, Takatsuki City, Osaka 569-8686, Japan; 2 Department of Pathology, Osaka Medical College, 2-7 Daigaku-machi, Takatsuki City, Osaka 569-8686, Japan

**Keywords:** Angiogenesis, Brain radiation necrosis, Inflammation, Platelet-derived growth factors, Platelet-derived growth factor receptors

## Abstract

**Background:**

Brain radiation necrosis (RN) occurring after radiotherapy is a serious complication. We and others have performed several treatments for RN, using anticoagulants, corticosteroids, surgical resection and bevacizumab. However, the mechanisms underlying RN have not yet been completely elucidated. For more than a decade, platelet-derived growth factors (PDGFs) and their receptors (PDGFRs) have been extensively studied in many biological processes. These proteins influence a wide range of biological responses and participate in many normal and pathological conditions. In this study, we demonstrated that PDGF isoforms (PDGF-A, B, C, and D) and PDGFRs (PDGFR-α and β) are involved in the pathogenesis of human brain RN. We speculated on their roles, with a focus on their potential involvement in angiogenesis and inflammation in RN.

**Methods:**

Seven surgical specimens of RN, obtained from 2006 to 2013 at our department, were subjected to histopathological analyses and stained with hematoxylin and eosin. We qualitatively analyzed the protein expression of each isoform of PDGF by immunohistochemistry. We also examined their expression with double immunofluorescence.

**Results:**

All PDGFs were expressed in macrophages, microglia, and endothelial cells in the boundary of the core of RN, namely, the perinecrotic area (PN), as well as in undamaged brain tissue (UB). PDGF-C, D and PDGFR-α were also expressed in reactive astrocytes in PN. PDGFs and PDGFR-α were scarcely detected in UB, but PDGFR-β was specifically expressed in endothelial cells not only in PN but also in UB.

**Conclusions:**

PDGFs/PDGFRs play critical roles in angiogenesis and possibly in inflammation, and they contribute to the pathogenesis of RN, irrespective of the original tumor pathology and applied radiation modality. Treatments for the inhibition of PDGF-C, PDGF-D, and PDGFR-α may provide new approaches for the treatment of RN induced by common radiation therapies.

## Background

Higher radiation doses to tumors result in good local tumor control and improvement in overall survival. On the other hand, radiation necrosis (RN) in the brain occurring after radiotherapy for brain tumors as well as for head and neck cancers is a serious complication that decreases the quality of life in patients. The mechanisms underlying RN have not been completely elucidated. In a previous study we showed that RN specimens stained with hematoxylin and eosin (H&E) typically show marked angiogenesis, so-called telangiectasis, microbleeding, and interstitial edema, probably caused by leakage of plasma from leaky angiogenesis into the surrounding necrotic core—namely, the perinecrotic area (PN) [1].

We and others have applied several treatments for RN, such as anticoagulants, vitamin E, corticosteroids, and surgical resection [2-4]. The typical MRI of symptomatic RN from case 3 demonstrated rapid shrinkage of the perilesional edema after surgical treatment [see Additional file 1 and Table 1]. After surgical resection for the only enhanced lesion, the perilesional edema decreased rapidly compared with preoperative MRI. This rapid shrinkage of the perilesional edema after surgical treatment was also observed in other cases. In addition, bevacizumab, an antibody for vascular endothelial growth factor (VEGF), has recently shown promising effects on symptomatic brain RN and symptomatic pseudo-progression [5,6]. However, in some cases, treatment with bevacizumab was not sufficient to resolve RN. Some RN cases recurred as RN even after temporary remission by bevacizumab treatment [7].

**Table 1:** Clinical features of patients with symptomatic radiation necrosis

**Pt.**	**Age (y)**	**Sex**	**Original dis.**	**Radiation**^**a**^	**Resection area (lobe)**	**Duration**^**b**^	**Chemo**
1	46	F	SCC.	XRT (60 Gy), BNCT (13.9 Gy-Eq)	Temporal	7	MTX
2	78	M	Sal. Duc. Ca.	XRT (60 Gy), BNCT (13.9 Gy-Eq)	Frontotemporal	20	-
3	18	M	GBM	XRT (IMRT) (74 Gy)	Parietal	37	-
4	63	F	GBM	XRT (24 Gy), BNCT (13 Gy-Eq)	Frontoparietal	4	-
5	34	M	GBM	XRT (24 Gy), BNCT (13 Gy-Eq)	Frontal	6	-
6	56	F	GBM	Proton + XRT (total 90 Gy)	Temporoparietal	10	ACNU
7	46	F	Ade. Ca.	XRT (30 Gy), SRS (55 Gy, 65 Gy)	Frontal	32	Herceptin

*Pt,* patient; *y,* year; *F,* female; *M,* male; *Original dis,* original disease; *SCC*, Squamous cell carcinoma; *Sal. Duc. Ca,* salivary ductal carcinoma; *GBM,* glioblastoma; *Ade. Ca,* adenocarcinoma; *XRT,* X-ray radiation treatment; *IMRT* ,intensity modulated radiation therapy; *BNCT,* boron neutron capture therapy; *Proton,* proton beam therapy; *MTX,* methotrexate; *ACNU,* nimustin; *Herceptin,* trastuzumab;

^a^In Pt. 1 and 2, the temporal lobe was included in the irradiation field and in Pt. 3, 4, 5, and 6, local radiation therapy was administered. Pt. 7 had received whole brain irradiation and received SRS twice. In BNCT, the presented dose is the peak point dose for the normal brain.

^b^Months between termination of the last radiotherapy and onset of symptoms caused by radiation necrosis.

Recent experiments have shown that demyelination and damage of the normal vasculatures and the appearance of abnormal vasculatures around necrotic foci are major issues in the development of RN [8,9]. In addition, we previously reported that hypoxia-inducible factor 1α (HIF-1α) and VEGF are key molecules in RN [1]. In a later study, we tried to determine whether not only HIF-1α and VEGF, but also proinflammatory cytokines such as IL-1α, IL-6, TNF-α, and NFκB, might play significant roles in RN, since these cytokines were produced by CD68- and hGLUT5-positive microglia and/or macrophages accumulated in PN (in submission).

The platelet-derived growth factors (PDGFs) signaling pathway, which has been extensively studied and shown to play critical roles in many biological processes, is mediated through tyrosine kinase receptors (PDGFR-α, PDGFR-β) [10,11]. There are five members of the PDGF family: PDGF-A, B, and AB, and the recently discovered PDGF-C and D. So far, no heterodimers involving the PDGF-C and D chains have been described. PDGF-A binds only PDGFR-α, whereas PDGF-B activates PDGFR-α, αβ, and β. PDGF-A, B, and C activate PDGFR-α and αβ, while PDGF-D specifically binds to and activates its cognate receptor PDGFR-β. In other words, according to published data, PDGFR-α binds PDGF-A, B, AB, and C, whereas PDGFR-β binds PDGF-B and D [10,12,13].

In addition, PDGF-A and B are secreted in their active forms, while PDGF-C and D are secreted as inactive forms requiring activation for their function [14]. Interestingly, several reports have shown that the structure and biological function of PDGFs are quite similar to those of VEGF [15]. Therefore, the PDGF family is sometimes referred to as the VEGF family. Nevertheless, in recent years it was revealed that the angiogenic pathway induced by PDGF-C is, in large part, VEGF-independent [16].

Based on these findings, in this retrospective study we performed histopathological and immunohistochemical analyses on 7 human RN specimens from patients who we had treated surgically from 2006 to 2013 at our department. We here describe the findings common to all 7 of these specimens, and demonstrate which type of cells produce PDGFs and which type express the PDGFRs. We also evaluated the roles of PDGFs/PDGFRs in brain RN.

## Methods

### Case selection

Seven surgical specimens, obtained from 2006 to 2013, were submitted for histopathological analysis, staining with H&E, and immunohistochemistry. All the patients had received radiotherapy, including X-ray treatment (XRT), stereotactic radiosurgery (SRS), proton beam therapy, and boron neutron capture therapy (BNCT). The primary diseases were 4 glioblastomas, 2 head and neck cancers, and 1 metastatic brain tumor derived from breast cancer.

In this study we selected the area as radiation necrosis with extensive necrotic area with the boundary of extensive angiogenesis and edema, which is continuous to undamaged brain tissue, as mentioned in Background.

For the 2 patients with head and neck cancers, radiotherapy was used to treat the parotid lesions and the temporal lobe was included in the irradiation field. Therefore, there were no tumor cells in the brain, indicating pure brain RN. The patient characteristics are detailed in Table 1.

### Histological and immunohistochemical staining

Histological and immunohistochemical analyses were performed on paraffin sections in which we observed the presence of RN by H&E staining. Each section was immunostained with the following antibodies: PDGF-A (1:20; R&D Systems, USA), PDGF-B (1:20; Abcam, Japan), PDGF-C (1:100; R&D Systems), PDGF-D (1:50; R&D Systems), PDGFR-α (1:20; R&D Systems), and PDGFR-β (1:50; R&D Systems) (Table 2). We routinely use a pressure cooker for 4 minutes to retrieve all the antigens. Endogenous peroxidase was blocked with 0.03% hydrogen peroxide for 40 minutes at room temperature. We used the ABC technique (Vector Laboratories, USA) for all of these antigens, before DAB (3, 3′ diaminobenzidine tetrahydrochloride (Wako Pure Chemical Industries, Japan)). The sections were counterstained with hematoxylin 3G (Sakura Finetek, Japan) and mounted.

**Table 2:** List of primary antibodies used

**Antibody**	**Clone**	**Sources**	**Type**	**Dilution**
PDGF-A		R&D Systems, Minneapolis, MN	p/g	1:20
PDGF-B	MM0014-5 F66	Abcam Cambridge, MA	m/m	1:20
PDGF-C		R&D Systems, Minneapolis, MN	p/g	1:100
PDGF-D		R&D Systems, Minneapolis, MN	p/g	1:50
PDGFR-α		R&D Systems, Minneapolis, MN	p/g	1:20
PDGFR-β		R&D Systems, Minneapolis, MN	p/g	1:50
CD68	KP-1	Dako, Glostrup, Denmark	m/m	1:25
hGLUT5		IBL, Tokyo, Japan	p/r	1:50
GFAP	6 F2	Dako, Glostrup, Denmark	m/m	1:25
CD45	EP322Y	Eptomics, Burlingame, CA	m/r	1:50
CD31	JC70A	Dako, Glostrup, Denmark	m/m	1:20

*p/g* polyclonal goat; *p/r*, polyclonal rabbit; *m/r*, monoclonal rabbit; *m/m*, monoclonal mouse.

### Immunofluorescence

Double immunofluorescence was performed using the following antibody combinations: PDGF-C and GFAP (1:25; Dako, Denmark), CD68 (1:25; Epitomics, USA), hGLUT5 (1:50; IBL, Japan), or CD45 (1:50; Epitomics); PDGF-D and GFAP, CD68, hFLUT5, or CD45; PDGFR-α and GFAP, CD68, hGLUT5, or CD31 (1:20; Dako, Denmark); and PDGFR-β and GFAP, CD68, hGLUT5, or CD31.

GFAP, CD68, hGLUT5, CD45, and CD31 were adopted as markers for astrocytes, monocytes, microglia, lymphocytes, and endothelial cells, respectively. All sections were incubated with their respective antibodies for 24 hours with CD68, hGLUT5, and GFAP, and for 48 hours with PDGF-A, B, C, D, and PDGFR-α and β. Then, after washing the primary antibodies, Alexa Fluor 488 (1:25; Molecular Probes, USA) or Alexa Fluor 546 (1:25; Molecular Probes) was used (Table 3). Finally, the sections were examined using an LSM510 laser scanning confocal microscope (Carl Zeiss, Germany).

**Table 3:** Double immunofluorescence combinations

**Primary**	**Dilution**	**Secondary**	**Primary**	**Dilution**	**Secondary**
PDGF-C	1:50	F488	CD68	1:25	F546
PDGF-C	1:50	F488	hGLUT5	1:50	F546
PDGF-C	1:50	F488	GFAP	1:25	F546
PDGF-C	1:50	F488	CD45	1:50	F546
PDGF-D	1:20	F488	CD68	1:25	F546
PDGF-D	1:20	F488	hGLUT5	1:50	F546
PDGF-D	1:20	F488	GFAP	1:25	F546
PDGF-D	1:20	F488	CD45	1:50	F546
PDGFR-α	1:10	F488	CD68	1:25	F546
PDGFR-α	1:10	F488	hGLUT5	1:50	F546
PDGFR-α	1:10	F488	GFAP	1:25	F546
PDGFR-α	1:10	F488	CD31	1:20	F546
PDGFR-β	1:20	F488	CD68	1:25	F546
PDGFR-β	1:20	F488	hGLUT5	1:50	F546
PDGFR-β	1:20	F488	GFAP	1:25	F546
PDGFR-β	1:20	F488	CD31	1:20	F546

*Primary*, primary antibody; *Secondary*, secondary antibody; *F488*, Alexa Fluor 488; *F546*, Alexa Fluor 546.

### Statistical analysis

We assessed the frequency of expression of PDGFs semi-quantitatively by the following method. Five fields of each PDGF isoform in which abnormal angiogenesis was detected were randomly selected with a microscope. PDGF-positive mononuclear cells were counted. We observed 7 cases and, to reduce bias, used two observers to count the cells. One observer, who was blinded to the patients’ clinical and pathological information, evaluated the results of immunohistochemical staining. The ratios of PDGF-positive cells per total cells in each field were calculated, and we statistically analyzed the data with Steel-Dwass tests using JMP Pro 10 (SAS Institute, USA). The results revealed that PDGF-C and D showed higher frequency of expression than PDGF-A and B in PN. The difference was statistically significant.

### Ethical approval

This study was approved by an institutional committee of Osaka Medical College. The research was in compliance with the Helsinki Declaration.

## Results

### Expression of PDGFs

**Figure 1:**
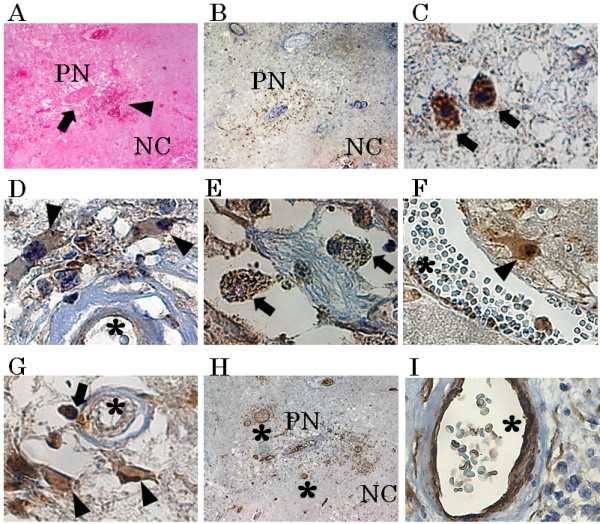
**Results of hematoxylin and eosin staining (H&E) and immunohistochemistry from case 1.** H&E staining **(A)** revealed a necrotic core (NC) and perinecrotic area (PN), including micro bleeding (**A**, arrowhead) and abnormal angiogenesis (**A**, arrow). Immunostaining results for PDGF-C are presented as a representative example **(B)**. PDGF-C **(C and D)**, **D (E and F)** and PDGFR-α **(G)** were produced by monocytic cells (**C**, **E**, **G**, arrow) and reactive astrocytic cells (**D**, **F**, **G**, arrowhead) in PN. On the other hand, PDGFR-β **(H and I)** was expressed mainly in endothelial cells (H and I*). There was partially nonspecific staining in NC **(B)** or around blood vessels **(I)**. Original magnification, **A**, **B** and **H** × 40, **C**, **D**, **E**, **F**, **G** and **I** × 200.

**Figure 2:**
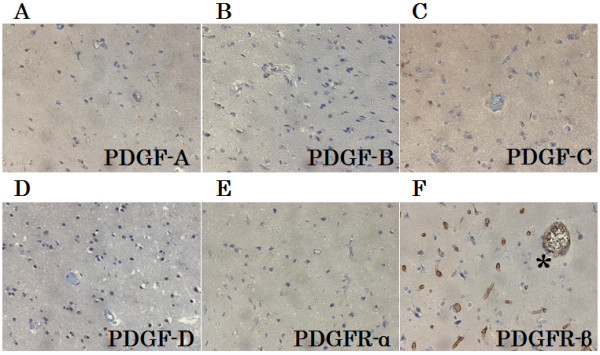
**Representative results of immunostaining of undamaged brain tissue (UB).** PDGF-**A**, **B**, **C**, **D** and PDGFR-α were scarcely detectable in UB **(A through E)**. PDGFR-β **(F)** was specifically expressed in endothelial cells in UB. Many normal cerebral blood vessels stained with PDGFR-β (F *) were detected in UB. Original magnification, ×200.

Figure 1 shows the results of H&E staining and immunohistochemistry from case 1. H&E staining revealed a necrotic core (NC) (Figure 1A. NC) and PN (Figure 1A. PN), in which micro bleeding (Figure 1A. arrowhead) and abnormal angiogenesis (Figure 1A. arrow) were confirmed. PDGF-A, B, C, and D-positive cells were detected in PN. The results of immunostaining for PDGF-C are shown as a typical example of these distribution analyses (Figure 1B, C, D). Morphologically, PDGF-A and B were produced by some monocytic cells [see Additional file 2] in PN. On the other hand, PDGF-C and D (Figure 1E, F) were produced by many monocytic cells (arrows in Figure 1C, E), reactive astrocytic cells (arrowheads in Figure 1D, F), and endothelial cells (Figure 1D*). PDGF-A, B, C, and D were scarcely detectable in UB (Figure 2).

These relationships among the expression of PDGFs are summarized in Table 4. These relationships were also confirmed with other specimens [see Additional file 3].

**Table 4:** Expression of PDGFs/PDGFRs in two areas of the brain

	**UB**			**PN**		
	**Mono**	**Astro**	**Endo**	**Mono**	**Astro**	**Endo**
PDGF-A	-	-	-	+	-	+
PDGF-B	-	-	-	+	-	+
PDGF-C	-	-	-	+	+	+
PDGF-D	-	-	-	+	+	+
PDGFR-α	-	-	-	+	+	+
PDGFR-β	-	-	+	-	-	+

*UB*, undamaged brain area; *PN*, perinecrotic area; *Mono*, monocytes, including macrophages, microglia and lymphocytes *Astro*, reactive astrocytes; *Endo*, endothelial cells ; *-*, not expressed; *+*, expressed.

**Figure 3:**
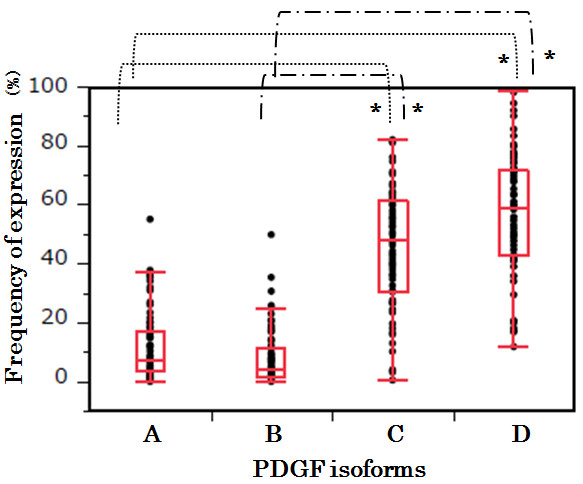
**Frequency of expression.** We assessed the frequency of expression of PDGFs semi-quantitatively by the following method. Five fields of each PDGF isoform, in which abnormal angiogenesis were detected, were randomly selected with a microscope. The PDGF-positive mononuclear cells were counted. We observed all 7 cases and performed the counting using two observers to reduce bias. One observer, who was blind to the patients’ clinical and pathological information, evaluated the results of the immunohistochemical staining. The ratios of PDGF-positive cells to total cells in each field were calculated and were statistically analyzed using Steel-Dwass tests with JMP Pro 10 (SAS Institute Inc., Cary, NC, USA). Statistical analysis revealed that PDGF-C and D showed higher frequency of expression in the PN specimens than did PDGF-A and B. The difference was statistically significant (*p < 0.0001, Steel-Dwass test).

Our statistical analysis revealed that PDGF-C and D showed higher frequencies of expression than PDGF-A and B in PN. The difference was statistically significant (p < 0.0001, Steel-Dwass test) (Figure 3). We also grouped the cases into a GBM group (cases 3, 4, 5, 6) and non-GBM group (cases 1, 2, 7) and analyzed the differences in protein expression between them. No statistically significant differences in the expression of any of the isoforms were observed between the two groups by the Steel-Dwass test [see Additional file 4]. Therefore, we considered that these primary diseases did not affect the expression of PDGFs.

**Figure 4:**
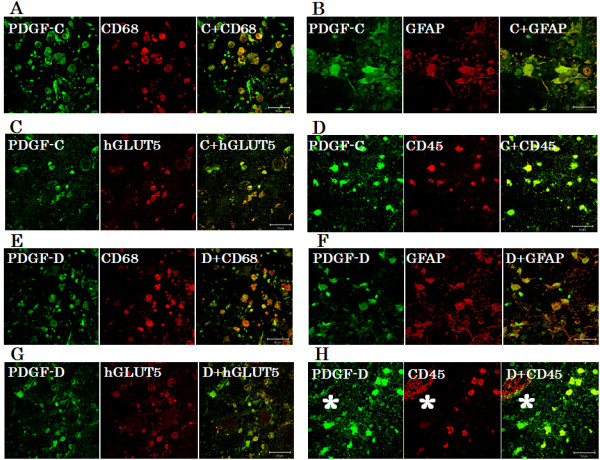
**Double immunofluorescence staining.** The results of double immunofluorescence staining from case 1 revealed that PDGF-C or D-positive cells were merged with many CD68 **(A, E)**, GFAP **(B, F)**, hGLUT5 **(C, G)**, and CD45 **(D, H)** -positive cells in PN. Some PDGF-C or D-positive cells did not express CD68, GFAP, hGLUT5 or CD45 and vice versa. Endothelial cells (*) were nonspecifically stained with secondary fluorescence antibody. The scale bar represents 50 μm.

Double immunofluorescence from case 1 revealed that PDGF-C or D-positive cells were merged with many cells positive for CD68 (Figure 4A, E), GFAP (Figure 4B, F), hGLUT5 (Figure 4C, G), and CD45 (Figure 4D, H).

H&E staining, immunohistochemistry, and double immunofluorescence also showed similar tendencies in other specimens with symptomatic RN [see Additional files 3, and 5].

### Expression of PDGFRs

PDGFR-α was expressed in endothelial cells (Figure 1G*), monocytic cells (Figure 1G arrow), and reactive astrocytic cells (Figure 1G, arrowhead) in PN. PDGFR-β was expressed mainly in endothelial cells (Figure 1H, I*). PDGFR-α was not expressed in any types of cells in UB (Figure 2E), but PDGFR-β was detected in endothelial cells in both PN and UB (Figure 2F).

**Figure 5:**
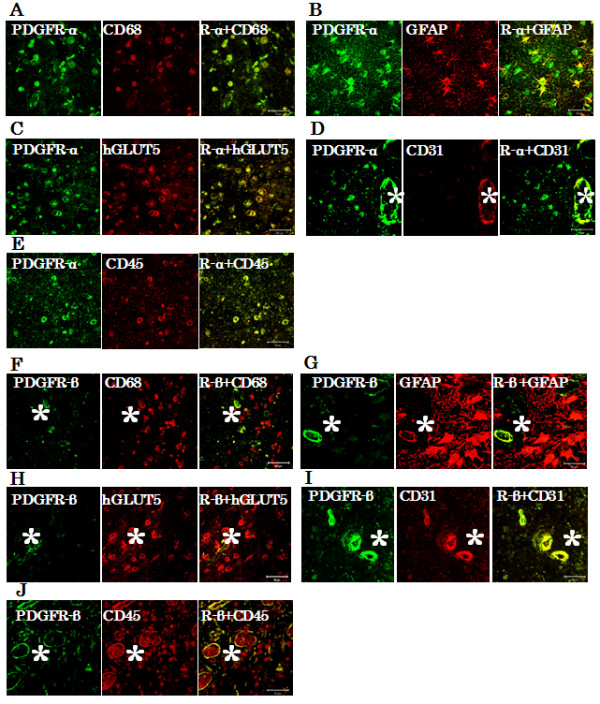
**Double immunofluorescence staining.** Double immunofluorescence staining from case 1 revealed that PDGFR-α and β were strongly expressed in CD31-positive cells in PN **(D and I)**. PDGFR-α positive cells were merged with many cells positive for CD68 **(A)**, GFAP **(B)**, hGLUT5 **(C)**, and CD45 **(E)**. PDGFR-β-positive cells merged specifically with endothelial cells (F, G, H, I and J, *). Endothelial cells (*) were nonspecifically stained with secondary fluorescence antibody. The scale bar represents 50 μm.

Double immunofluorescence revealed that PDGFR-α and β were strongly expressed in CD31-positive cells (Figure 5D, I). PDGFR-β-positive cells were merged specifically with endothelial cells (Figure 5F, G, H, I and J, *), but PDGFR-α-positive cells were merged with cells positive for CD68 (Figure 5A), GFAP (Figure 5B), hGLUT5 (Figure 5C), and CD45 (Figure 5E) in PN.

These findings from case 1 were confirmed in other specimens with symptomatic RN [see Additional file 6].

Double immunofluorescence revealed partially nonspecific staining, especially in endothelial cells (Figures 4, and 5*). In cases where immunofluorescence was performed with GFAP alone, vascular endothelial cells were not stained [see Additional file 7]. These findings were also observed in other specimens.

## Discussion

PDGFs are a group of multifunctional proteins with a wide variety of effects. They have important physiologic functions in embryonic and organ development, have been implicated in a wide variety of pathological processes, including proliferation, differentiation, and fibrogenesis, and are essential for the stability of normal blood vessel formation [16-19]. However, the overexpression of PDGFs has adverse effects. Previous studies also have demonstrated that various cell types, including macrophages, fibroblasts, pericytes, and capillary endothelial cells, express PDGFs [20,21]. Deuel et al. also reported that a macrophage-derived PDGF induces chemotaxis and the proliferation of monocytes and fibroblasts during inflammation and wound repair [22].

This is the first study to explore the expression of PDGF isoforms and PDGFRs in human brain RN. Our results have shown that all PDGFs and PDGFRs were expressed in brain RN, and that PDGFs and PDGFRα were primarily expressed by macrophages, microglia, reactive astrocytes, lymphocytes, and endothelial cells in PN. These findings suggest that the activation of PDGFs is coincident with inflammation, angiogenesis, and fibrogenesis in the pathophysiology of RN.

Our recent study revealed that CD45-positive lymphocytes expressing CXCR4 might be drawn into PN from peripheral blood by chemotaxis, but they do not express proinflammatory cytokines, and their roles in RN remain unclear (submitted for publication). However, in the present study, CD45-positive lymphocytes produced PDGF-C and -D. These results suggest that CD45-positive lymphocytes in PN do not produce proinflammatory cytokines but may play significant indirect roles in angiogenesis and/or inflammation.

The highest differences of expression among PDGFs on brain RN were observed in PDGF-C and D (Figure 3 and Additional file 4). In this study, the expressions of PDGF-C and D were significantly higher than the expressions of PDGF-A and B in PN. Our current immunohistochemical study has further revealed that inflammatory cells, including macrophages, microglia, and even lymphocytes, were gathered in PN and produced PDGF-C and D. These mononuclear cells are known to play important roles in wound healing and inflammatory disease by producing a variety of growth factors and cytokines [23,24]. In our recent study, these mononuclear cells produced inflammatory cytokines (IL-1α, IL-6, TNF-α, NFκB) (submitted for publication). In the present study, these cells also produced PDGF-C and D. Therefore the activation of PDGF-C and D is coincident with inflammation as well as angiogenesis. These findings suggest that PDGF-C and D are involved in multiple aspects of brain RN.

The present and previous reports have revealed that the differential expression of PDGFs has also been seen in pathological conditions other than RN. In the aortic ring outgrowth assay, PDGF-C mediated significantly increased outgrowth, comparable to the levels mediated by VEGF and PDGF-A and B [25]. The angiogenic activity of PDGF-C in vivo is more potent than that of PDGF-A, AB or B [26]. PDGF-D also has been shown to stimulate angiogenesis and to play a critical role in wound healing [21,27,28].

Li et al. found that PDGF-D is a potent transforming and angiogenic growth factor for NIH/3 T3 cells, and that the transformed cells also induce VEGF expression [28]. Zhao et al. also found that inhibition of PDGF-D leads to decreased cell invasion in gastric cancer, partly through the regulation of VEGF [29]. In our study, many reactive astrocytes produced PDGF-C and D and expressed PDGFR-α, but these cells did not express PDGFR-β. These results established that PDGF-C and D play roles in angiogenesis and inflammation through autocrine and paracrine stimulation. Although the functions of these isoforms of PDGFs on cells are similar in many respects, each isoform might play different roles in different cell types via various receptors and pathways.

Previously, it was reported that several types of cells participate in angiogenesis and inflammation in brain RN [1,5,6]. But the underlying mechanisms have not been completely elucidated. We desperately need to know why different types of cells, including macrophages, microglia, lymphocytes, and astrocytes, acquire the capacity for differentiation, producing inflammatory cytokines and growth factors under certain pathological conditions. Ungvari et al. reported that γ-irradiated cerebromicrovascular endothelial cells acquired a senescence-associated secretory phenotype (SASP) characterized by the upregulation of proinflammatory cytokines and chemokines [30]. Our results suggest that several types of cells that survived irradiation in PN acquired SASP, and that this mechanism may be a key process in brain RN.

In this study, we performed retrospective analysis with clinical specimens of symptomatic RN and revealed that PDGFs/PSGFRs were involved in RN. However, this analysis covers just one aspect of RN. It is impossible to determine whether PDGFs exacerbate RN or rather are produced as a byproduct of RN. Also, we cannot speculate as to the dose–response relationship or the time course of the expression of PDGFs and their receptors in RN. These questions will be answered if a reproducible animal model of RN can be established.

## Conclusions

In conclusion, PDGFs/PDGFRs play critical roles in angiogenesis and possibly in inflammation, and they contribute to the pathogenesis of RN, irrespective of the original tumor pathology and applied radiation modality. Moreover, the autocrine or paracrine signaling of PDGFs also plays crucial roles in aggressive angiogenesis and inflammation in RN. PDGF-C, PDGF-D and PDGFR-α have clinical importance, because PDGFR-β was expressed even in UB. Treatments to inhibit PDGF-C and D, or to inhibit PDGF-C and D in combination with PDGFR-α with a kinase inhibitor, may provide new approaches for RN induced by common radiation therapies, including XRT, SRS and BNCT.

## Abbreviations

RN: Radiation necrosis; PDGFs: Platelet-derived growth factors; PDGFRs: Platelet-derived growth factor receptors; H&E: Hematoxylin and eosin; PN: Perinecrotic area; UB: Undamaged brain tissue; VEGF: Vascular endothelial growth factor; HIF-1α: Hypoxia-inducible factor 1α; XRT: X-ray treatment; SRS: Stereotactic radiosurgery; Proton: Proton beam therapy; BNCT: Boron neutron capture therapy; NC: Necrotic core; SASP: Senescence-associated secretory phenotype; CTLs: Cytotoxic T-lymphocytes.

## Competing interests

The authors declare that they have no competing interests.

## Authors’ contributions

TM carried out the statistical analysis and drafted the manuscript. S-IM conceived of the study, participated in its design and coordination, and helped to draft the manuscript. TT, NN, MF, HK, EY, SK, and TK participated in the study design and coordination and helped to draft the manuscript. All authors read and approved the final manuscript.

## Supplementary Material

Additional file 1 **Typical MRI of symptomatic radiation necrosis from case 3.** Gd-enhanced T1 MRI just prior to excision of necrotic foci (A). Gd-enhanced T1 MRI 2 weeks after surgery (A’). FLAIR MRI just prior to excision of necrotic foci (B). FLAIR MRI, 2 weeks after surgery (B’). After surgical resection of the only enhanced lesion, perilesional edema was decreased compared with preoperative MRI. Click here for file

Additional file 2 **Representative immunohistochemistry from case 1.** Immunostaining revealed the necrotic core (A, D NC) and perinecrotic area (A, D PN). PDGF-A (A, B, C) and PDGF-B (D, E) were produced by some monocytic cells (B, E arrow) and endothelial cells (C, E*) in PN. Original magnification, A, D × 40, B, C, E × 200. Click here for file

Additional file 3 **H&E staining and immunohistochemistry from case 3.** H&E staining (A) and immunohistochemistry (B through O) from case 3, showing NC and PN. PDGF-A (B, C) and PDGF-B (D, E) were produced by some monocytic cells (arrows in C, E) in PN. In contrast, PDGF-C (F, G, H) and PDGF-D (I, J) were produced by many monocytic cells (arrows in G, H, J), reactive astrocytic cells (arrowheads in G, J), and endothelial cells (H, J*). PDGFR-α (K, L, M) was expressed in monocytic cells (L, arrow), reactive astrocytic cells (L, arrowhead) and endothelial cells (M*) in PN. PDGFR-β (N, O) was expressed mainly in endothelial cells (O*). Original magnification, A, B, D, F, I, K, N × 40, C, E, G, H, J, L, M, O × 200. Click here for file

Additional file 4 **Frequency of expression of PDGFs in the GBM group and non-GBM group.** We assessed the frequency of expression of PDGFs semi-quantitatively. In the GBM group (cases 3, 4, 5, 6) and non-GBM group (cases 1, 2, 7), there was no apparent statistical significance in expression of each isoform (A, B, C, D). Click here for file

Additional file 5 **Double immunofluorescence staining results from case 3.** Double immunofluorescence staining from case 3 revealed that PDGF-C or D-positive cells were merged with many CD68, hGLUT5, CD45 and GFAP-positive cells. Endothelial cells (*) were nonspecifically stained with secondary fluorescence antibody. The scale bar represents 50 μm. Click here for file

Additional file 6 **Double immunofluorescence staining results from case 3.** Double immunofluorescence staining of the specimen from case 3 revealed that PDGFR-α and β were strongly expressed in CD31-positive cells (D and I). PDGFR-α-positive cells were merged with many cells positive for CD68 (A), GFAP (B), hGLUT5 (C), and CD45 (E). PDGFR-β-positive cells were merged specifically with endothelial cells (F thorough J). Endothelial cells (*) were nonspecifically stained with secondary fluorescence antibody. The scale bar represents 50 μm. Click here for file

Additional file 7 **Immunofluorescence staining from consecutive specimens from case 1 and 3.** Immunofluorescence staining of consecutive specimens from case 1 (A, B) and 3 (C, D) showed positivity for PDGFR-β (A) or GFAP (B). PDGFR-β (A) was not observable at an excitation wavelength of 561 nm but was apparent at 499 nm in endothelial cells (*). On the other hand, GFAP (B) was observed only at an excitation wavelength of 561 nm in reactive astrocytes. The scale bar represents 50 μm. Click here for file
