# Supplementary figures and images for: The roles of platelet-derived growth factors and their receptors in brain radiation necrosis
Source: Radiat Oncol. 2014 Feb 11;9:51. doi: 10.1186/1748-717X-9-51 (PMC3927833; doi:10.1186/1748-717X-9-51)

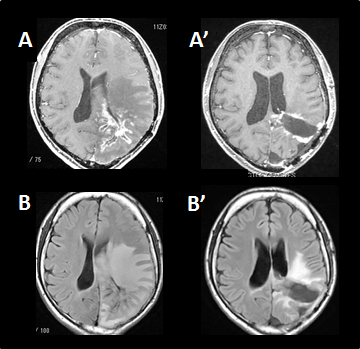

Supplement: Additional file 1 — Typical MRI of symptomatic radiation necrosis from case 3. Gd-enhanced T1 MRI just prior to excision of necrotic foci (A). Gd-enhanced T1 MRI 2 weeks after surgery (A’). FLAIR MRI just prior to excision of necrotic foci (B). FLAIR MRI, 2 weeks after surgery (B’). After surgical resection of the only enhanced lesion, perilesional edema was decreased compared with preoperative MRI. [file 1748-717X-9-51-S1.tiff]

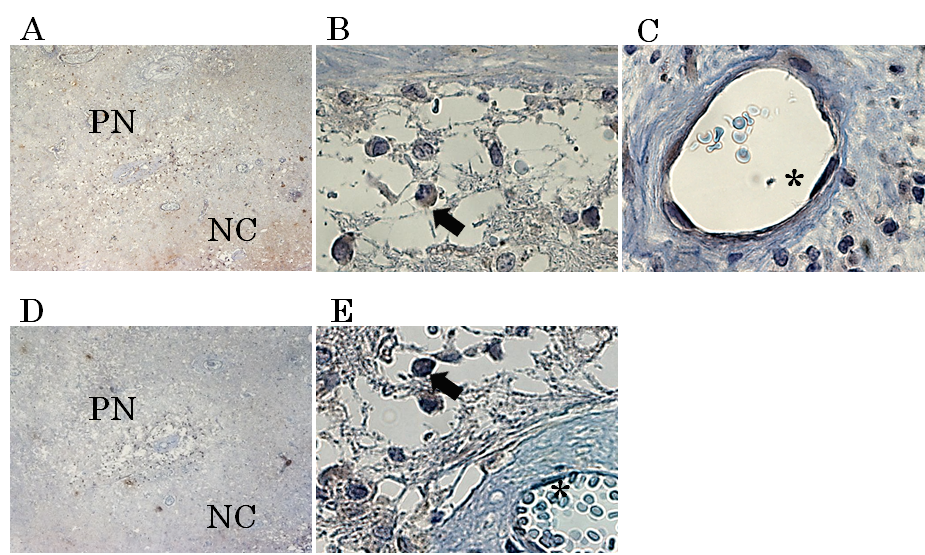

Supplement: Additional file 2 — Representative immunohistochemistry from case 1. Immunostaining revealed the necrotic core (A, D NC) and perinecrotic area (A, D PN). PDGF-A (A, B, C) and PDGF-B (D, E) were produced by some monocytic cells (B, E arrow) and endothelial cells (C, E*) in PN. Original magnification, A, D × 40, B, C, E × 200. [file 1748-717X-9-51-S2.tiff]

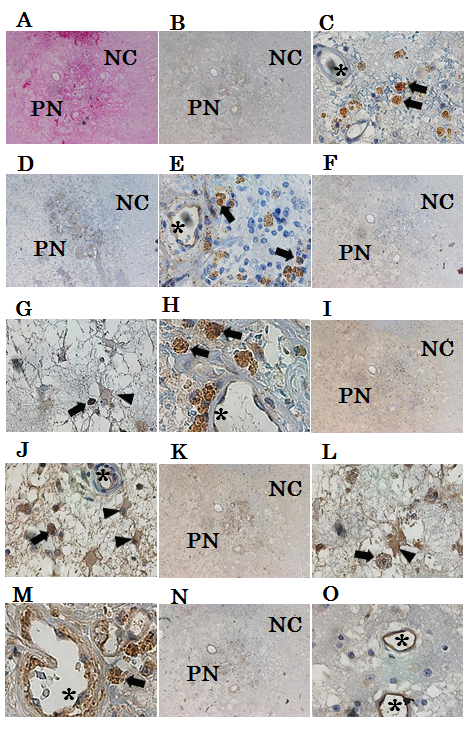

Supplement: Additional file 3 — H&E staining and immunohistochemistry from case 3. H&E staining (A) and immunohistochemistry (B through O) from case 3, showing NC and PN. PDGF-A (B, C) and PDGF-B (D, E) were produced by some monocytic cells (arrows in C, E) in PN. In contrast, PDGF-C (F, G, H) and PDGF-D (I, J) were produced by many monocytic cells (arrows in G, H, J), reactive astrocytic cells (arrowheads in G, J), and endothelial cells (H, J*). PDGFR-α (K, L, M) was expressed in monocytic cells (L, arrow), reactive astrocytic cells (L, arrowhead) and endothelial cells (M*) in PN. PDGFR-β (N, O) was expressed mainly in endothelial cells (O*). Original magnification, A, B, D, F, I, K, N × 40, C, E, G, H, J, L, M, O × 200. [file 1748-717X-9-51-S3.tiff]

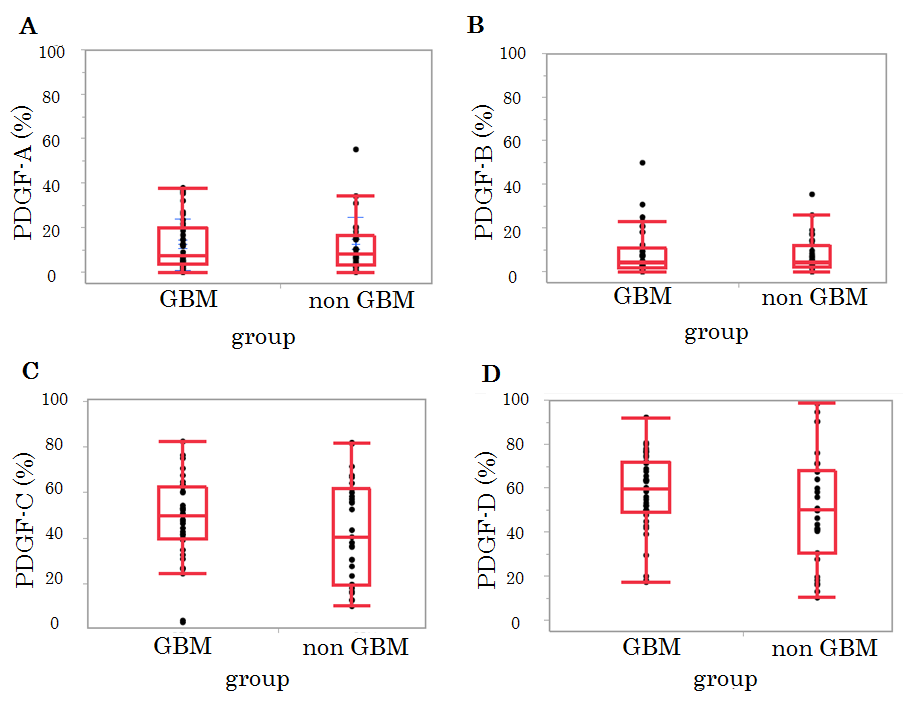

Supplement: Additional file 4 — Frequency of expression of PDGFs in the GBM group and non-GBM group. We assessed the frequency of expression of PDGFs semi-quantitatively. In the GBM group (cases 3, 4, 5, 6) and non-GBM group (cases 1, 2, 7), there was no apparent statistical significance in expression of each isoform (A, B, C, D). [file 1748-717X-9-51-S4.tiff]

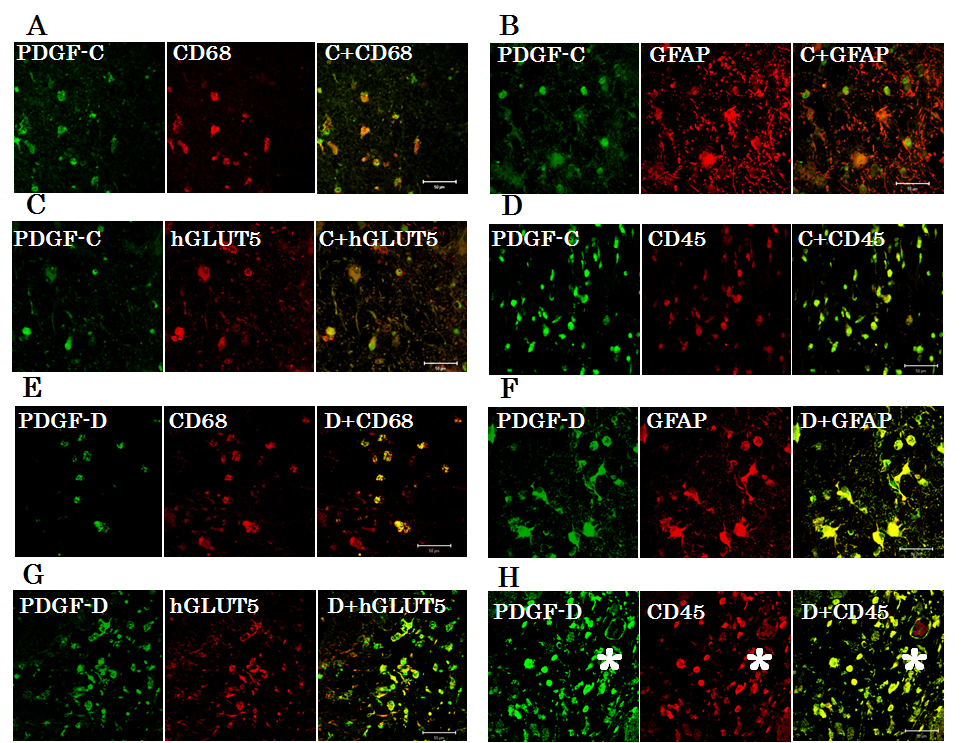

Supplement: Additional file 5 — Double immunofluorescence staining results from case 3. Double immunofluorescence staining from case 3 revealed that PDGF-C or D-positive cells were merged with many CD68, hGLUT5, CD45 and GFAP-positive cells. Endothelial cells (*) were nonspecifically stained with secondary fluorescence antibody. The scale bar represents 50 μm. [file 1748-717X-9-51-S5.tiff]

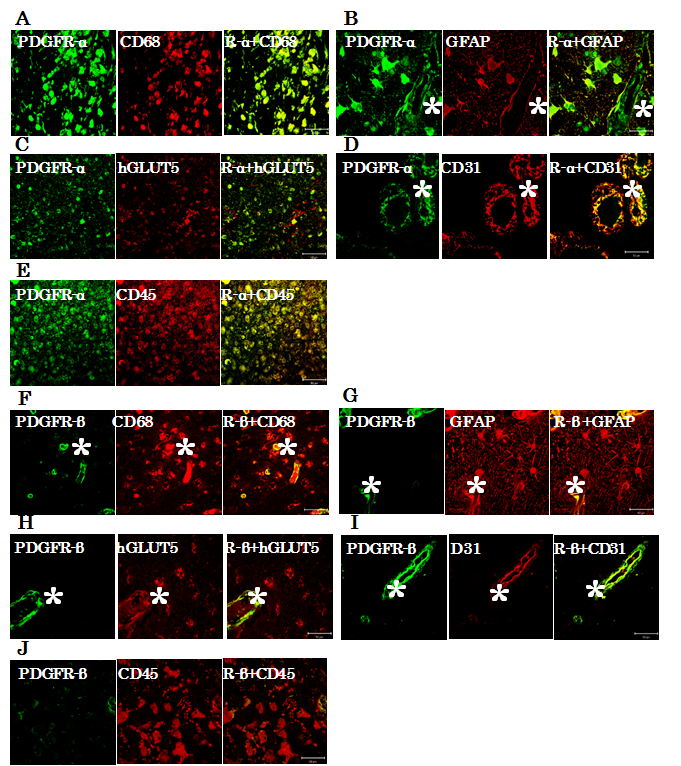

Supplement: Additional file 6 — Double immunofluorescence staining results from case 3. Double immunofluorescence staining of the specimen from case 3 revealed that PDGFR-α and β were strongly expressed in CD31-positive cells (D and I). PDGFR-α-positive cells were merged with many cells positive for CD68 (A), GFAP (B), hGLUT5 (C), and CD45 (E). PDGFR-β-positive cells were merged specifically with endothelial cells (F thorough J). Endothelial cells (*) were nonspecifically stained with secondary fluorescence antibody. The scale bar represents 50 μm. [file 1748-717X-9-51-S6.tiff]

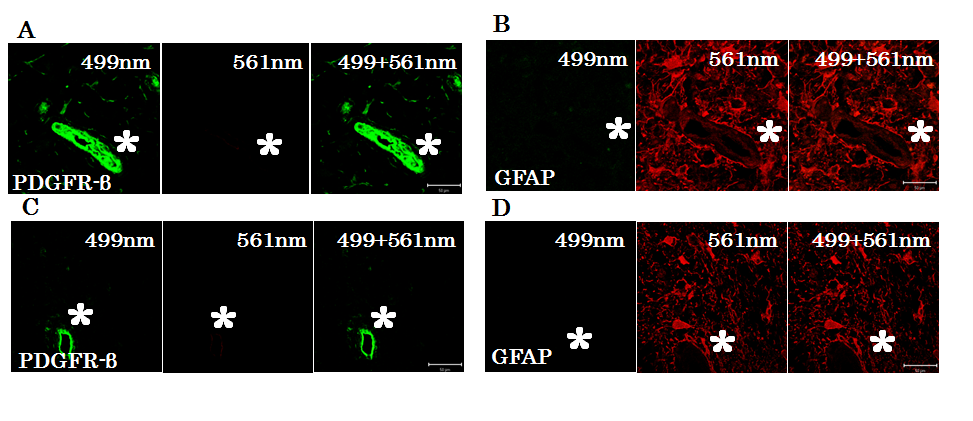

Supplement: Additional file 7 — Immunofluorescence staining from consecutive specimens from case 1 and 3. Immunofluorescence staining of consecutive specimens from case 1 (A, B) and 3 (C, D) showed positivity for PDGFR-β (A) or GFAP (B). PDGFR-β (A) was not observable at an excitation wavelength of 561 nm but was apparent at 499 nm in endothelial cells (*). On the other hand, GFAP (B) was observed only at an excitation wavelength of 561 nm in reactive astrocytes. The scale bar represents 50 μm. [file 1748-717X-9-51-S7.tiff]
